# Parenting Styles and Disordered Eating Among Youths: A Rapid Scoping Review

**DOI:** 10.3389/fpsyg.2021.802567

**Published:** 2022-01-27

**Authors:** Chloe Hampshire, Bérénice Mahoney, Sarah K. Davis

**Affiliations:** School of Psychology, University of Worcester, Worcester, United Kingdom

**Keywords:** youth, adolescents, parenting styles, authoritative, neglectful, disordered eating, family context

## Abstract

Youth is a critical period in the development of maladaptive eating behaviors. Previous systematic reviews suggest the etiological significance of parent-child relationships for the onset of disordered eating in youth, but less is known about the role of parenting styles. This rapid scoping review aimed to identify whether research supports the role of parenting styles in the development of disordered eating symptoms among youths. Sixteen studies, retrieved from three databases (PsycArticles, PsycInfo, and BASE), met the inclusion criteria: original studies, published in English, examined the effect of parenting styles (authoritative or neglectful) on cognitive (drives for thinness and body dissatisfaction) and behavioral (weight control behaviors) disordered eating outcomes, among young people up to 18 years of age. Studies supported an association between various youth disordered eating symptoms such as unhealthy weight control behaviors, and experiences of adverse parenting styles characterized by high levels of control and low levels of responsiveness. Associations between adverse parenting styles and youth disordered eating were frequently indirect and differed depending on the sex of the parent and offspring. Synthesis of findings was limited due to variation in the operationalization and measurement of parenting styles, family context and disordered eating across studies. Longitudinal and standardized research is required to better understand the dynamic associations between parenting styles and youth disordered eating. Implications for family-based care in clinical practice are discussed.

## Introduction

Disordered eating (DE) refers to maladaptive attitudes, behaviors, and cognitions related to eating and weight (Stice et al., 2010), and has been broadly applied to both clinical (Deas et al., 2011) and subclinical (National Health Service, 2018) populations. In the present review, DE encapsulates a subthreshold presentation of eating disorder (ED) symptomology in terms of severity and frequency (e.g., National Health Service, 2018). EDs – including anorexia nervosa, bulimia nervosa, binge eating disorder, other specified feeding and/or eating disorder, and avoidant/restrictive food intake disorder – are clinically diagnosed and involve the maladaptive use of food as a coping mechanism (National Health Service, 2021). DE may predict an ED later in life (Stice and Van Ryzin, 2019), with longitudinal research tracking the progression of subclinical symptoms into severe symptomology (Herle et al., 2020). Given that DE is a frequent antecedent of an ED diagnosis (for overview, see McClelland et al., 2020), evaluating the onset of DE is important for understanding the clinical course of EDs and, additionally, the improvement of health among subclinical populations. Further, youth (here defined as young people up to 18 years of age, e.g., Grogan et al., 2020) may be a critical period for DE onset. DE frequently manifests in teenage years (Elmasry and Khali, 2018), though children as young as eight have presented DE (Yilmaz Kafali et al., 2020). Engagement in weight control behaviours (WCB) is common in young people (estimated prevalence rate of 44.4%, Solmi et al., 2021) and occurs more frequently than a clinical diagnosis of an ED (Elmasry and Khali, 2018).

The influence of the parent-child relationship on offspring DE has been explored (Botta and Dumlao, 2009). One component of the parent-child relationship is parenting styles – the typical attitudes held, and behaviors exerted, by those occupying a parenting role (Baumrind, 1971, 1991). Whilst domain-specific behaviors are indicative of parenting practices (such as pressure to eat or weight criticism), parenting styles are characterized by the childrearing attitudes and behaviors presenting across a range of parenting contexts (Darling and Steinberg, 1993; Power, 2013). Parenting styles have been categorized into four typologies comprising of dimensional constructs of responsiveness and demandingness (Maccoby, 1994; Gorostiaga et al., 2019): authoritative, authoritarian, permissive, and neglectful (Baumrind, 1991). Putatively considered adverse parenting styles include authoritarian (exhibiting high demandingness and low responsiveness), permissive (exhibiting low demandingness and high responsiveness), and neglectful (exhibiting low demandingness and low responsiveness) typologies (Baumrind, 1991). Parenting styles demonstrating increased parental indifference (exhibiting low responsiveness to offspring needs) are associated with a range of ED diagnoses, including anorexia and bulimia nervosa (Grogan et al., 2020). Moreover, these “unfavorable” parenting styles have been etiologically implicated in later stages of illness (Stice and Van Ryzin, 2019). It is therefore critical to understand this association in earlier illness- and life-stages before subclinical symptoms develop into serious mental illness.

Parenting styles may be viewed as a process through which attachment with caregivers is established and maintained across development (Neppl et al., 2019). Parents’ fostering of a nurturing bond with infants is critical; optimal internal working models (predictively guiding future psychosocial functioning) are established by an early secure bond and are necessary for adaptive development (Bowlby, 1977). To this end, there is evidence to suggest insecure attachment styles are both directly (Jewell et al., 2016) and also indirectly (Gugliandolo et al., 2020), associated with youth DE. Indirect associations suggests theories of attachment offer an important theoretical lens for understanding parental risk pathways to the onset of DE in youth.

Within such risk pathways, youths’ experience of adverse parenting styles may be conceptualized as a non-abuse adverse life experience (ALE). Non-abuse ALEs refer to adverse experiences excluding abuse (e.g., sexual or physical) (Grogan et al., 2020), and are characterized by prolonged exposure wherein associated (frequently detrimental) impacts accumulate over time (Cavelzani et al., 2018). Youths’ experience of adverse parenting styles may be conceptualized as a non-abuse ALE given that parenting styles remain relatively stable throughout offspring youth development (Zhang et al., 2017), and have been associated with a variety of offspring psychopathological outcomes, including an ED in youth (Erriu et al., 2020) and adulthood (Grogan et al., 2020).

Although some studies have examined the impact of adverse parenting styles on DE symptoms in youth, the results lack consistency. Though demanding (McEwen and Flouri, 2009) and unaccepting (Kerr et al., 2021) parenting styles have been associated with youth DE, non-significant associations have also been reported between paternal (Zubatsky et al., 2015) and maternal (Berge et al., 2014) parenting styles and adolescent DE. As the parent-child relationship is dynamic throughout youth development – with parental influence decreasing in salience relative to other social influences (e.g., Branje, 2018) – parenting styles may not be as influential on offspring outcomes as youths mature. Empirical inconsistencies may also arise from variation in the way that parenting styles and DE have been operationalized and measured. For instance, studies inconsistently capture collective parental contributions within co-parenting contexts (Kuppens and Ceulemans, 2019) with some studies converging maternal and paternal parenting styles in measurement (e.g., Rozenblat et al., 2017). Further, unvalidated tools have been implemented (e.g., Zubatsky et al., 2015). Therefore, as the literature on parenting styles is patterned by conceptual, methodological, and empirical inconsistency, it is necessary to conduct a review (Aromataris and Pearson, 2014).

Previous systematic reviews have evaluated the etiological significance of the parent-child relationship for the onset for both clinical and subclinical maladaptive eating behaviors in youth. Insecure attachment styles (Jewell et al., 2016) and dysfunctional family systems (Langdon-Daly and Serpell, 2017) have been associated with DE and ED symptom presentation among teenagers, respectively. However, to date, a focused systematic search has not been conducted for parenting styles and maladaptive eating behaviors among youth samples. For the present rapid scoping review, a symptom-based approach is undertaken due to (1) the relevance of DE to youth maladaptive eating behaviors (Elmasry and Khali, 2018; Yilmaz Kafali et al., 2020) and (2) the illness progression of DE into an ED (Stice and Van Ryzin, 2019). In sum, the current review aims to establish to what extent the evidence suggests that youths with experience of adverse parenting styles present with DE symptoms by systematically reviewing and methodologically evaluating all relevant literature in the field.

## Methods

**FIGURE 1:**
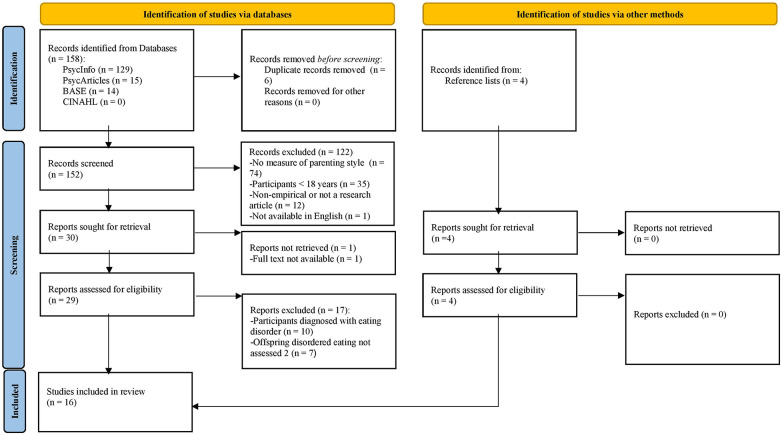
PRISMA 2020 flow diagram for new systematic reviews which included searches of databases and other sources (Page et al., 2021).

In this rapid scoping review, a systematic search of the literature was conducted (Figure 1). SD & CH decided on the scope and focus of the review and selection of search terms and inclusion criteria. Given that the present study is not a full systematic review, a single-reviewer coding and analysis of studies was deemed appropriate and was undertaken by CH in discussion with SD, as per Gartlehner et al. (2020).

### Search Strategy

PsycArticles, PsycInfo, and CINAHL were searched in March 2021 to gather primary published studies. The BASE database was also searched for gray literature for additional relevant sources. *Parenting style* and *teenage* or *childhood* (with synonyms) were used as search terms, combined with the following (including their abbreviations, synonyms, and derivatives): *disordered eating* and *maladaptive eating*. Full search strings are included in the Appendix. Manual searches of reference lists of relevant articles were conducted to source potential studies not included in original database searches.

### Inclusion and Exclusion Criteria

Included studies met the following requirements: (1) included a youth sample (up to 18 years of age, e.g., Grogan et al., 2020); (2) incorporated a measure of parenting style; (3) participants had not been diagnosed with an ED; (4) were empirical and not a theoretical paper or meta-analysis; (5) quantitative; (6) published after 1980, corresponding with the publication of the DSM-III (e.g., Grogan et al., 2020); and (7) available in English.

Further to criteria (2); studies solely measuring parenting behaviors relating to food or exercise, such as food restriction (e.g., Loth et al., 2014), were excluded from the review as these are indicative of domain-specific parenting practices (e.g., Darling and Steinberg, 1993) and not of parenting styles presenting across a range of situations (e.g., Baumrind, 1971). Furthermore, (5); as a systematic search has not been conducted for parenting styles and youth populations in the ED field, there were no restrictions on the type of study design included in order to capture all relevant research.

### Data Extraction and Synthesis

One hundred and fifty eight studies were identified from initial database searches. Following the removal of duplicates and screening, 122 studies were excluded, primarily for not including a measure of parenting style or for using an adult sample. Full-texts of the remaining 34 studies were evaluated, yielding 16 eligible studies for inclusion. Studies were excluded for using participants with an ED diagnosis or not assessing offspring DE.

Data from selected studies were extracted by CH and included studies’ author; year; country; design; sample characteristics; measure of parenting style and DE; parents assessed; risk of bias; and findings (Table 1). Given the heterogenous selection of DE outcomes and measures of parenting styles, a narrative synthesis was deemed the most appropriate form of analysis.

**TABLE 1:** Characteristics of cross-sectional and longitudinal studies analyzing the effect of parenting styles on youth presentation of disordered eating.

								
References	Study design	Country of origin	Sample characteristics	Parenting style measure	Parents assessed	Disordered eating measure	Relevant findings	Quality appraisal
								
Berge et al., 2014	Cross-sectional	United States	*N* = 2,793 (1,307 females) *M* = 14.4 years; S.D. = 2.0; range = 11–19	Family assessment device	Mother and father	Eating and activity in teens (EAT-26)	Youths reporting greater parental psychological control were more likely to engage in dieting and DEB behaviors. Psychologically controlling mothers were most associated with more DEB in males (extreme WCB) and all DEB in females (dieting, binge eating, unhealthy, and extreme WCB). For females, those with experience of higher perceived paternal psychological control were more likely to engage in all DE behaviors.	Moderate
Blodgett Salafia et al., 2009	Longitudinal attrition N/A	United States	*N* = 131 (73 females) *M* = 11.65 years; S.D. = 0.51; range = 11–13 years	Psychological control scale-youth self-report	Mother	Eating Disorder Inventory (EDI-1)	For both male and female participants, reported maternal psychological control predicted bulimic symptoms 2 years later. However, this association was indirect and mediated by adolescents’ lowered self-competence.	Weak
Blodgett Salafia et al., 2007	Longitudinal attrition N/A	United States	*N* = 73 (73 female) *M* = 11.59 years; S.D. = 0.52; range = 11–13 years	Psychological control scale youth report democracy scale parental knowledge scale	Mother	Eating Disorder Inventory (EDI-1)	The mother-youth relationship was important. Girls reporting their mothers’ undemocratic parenting style, restriction of psychological autonomy, and less knowledge of daily activities and social interactions were more likely to engage in DEB (dieting) and attitudes (drive for thinness and body dissatisfaction) 2 years later. However, this relationship was indirect, and fully mediated by internalized psychological distress.	Moderate
Fonseca et al., 2002	Cross-sectional	Portugal	*N* = 9,042 (4,625 female) Range = 12–18 years	Voice of Connecticut youth	Converged	Voice of Connecticut youth	Parenting style risk factors for engaging in DEB (extreme dieting) among males were reports of high parental supervision and monitoring. In contrast, there were no significant parenting risk factors associated with females.	Weak
Furnham and Adam-Saib, 2000	Cross-sectional	United Kingdom	*N* = 168 (168 female) Range = 15–17 years	Parental bonding instrument	Mother and father	Eating attitudes test (EAT-26)	Many findings were non-significant. Parental overprotection scores were not significantly associated with EAT scores across any groups. However, both maternal and paternal responsiveness negatively correlated with body satisfaction. Further, socio-cultural factors influenced the development of DE symptoms, with the British-Asian sample reporting higher EAT scores and parental overprotectiveness compared to white participants.	Weak
Hautala et al., 2011	Longitudinal T0 *N* = N/A T1 *N* = 1,891 T2 *N* = 935	Finland	*N* = 722 (535 female) *M* = 14.9 years. Divided into reported eating disturbance (*n* = 208), and non-symptomatic controls (*n* = 514)	Parental Bonding Instrument	Mother and father	SCOFF (Finnish translation)	Due to attrition, analyses were conducted on females. Parenting styles were associated with both onset of and remission from DE. Paternal overprotection alone was significantly associated with onset of DE; maternal parenting style was not. However, only maternal care uniquely predicted the recurrence of DE in adolescents. Further, prolonged DE symptoms were associated less caring and more controlling parenting styles. In terms of remission, “good enough” parenting was sufficient to remit female participants from symptoms.	Moderate
Hochgraf et al., 2017	Cross-sectional	United States	*N* = 699 (351 female) Range = 11–12 years	Parental Warmth report	Mother and father	Child’s eating habits and body self-image scale	Both maternal and paternal hostility were independently associated with youth DE. Further, an additive effect was observed as associations between youth DE and parenting styles were amplified when both parents were high in hostility. However, differential implications of parenting styles were observed, as paternal hostility was associated with more severe DE.	Weak
Kerr et al., 2021	Longitudinal T0 *N* = 4,950 T1 *N* = 4,950	United States	T0 *N* = 4,950 (2,370 female) Range = 9–10 years	Children’s report of parent behavior inventory	Mother and father	Kiddie schedule for affective disorders and Schizophrenia	Effects of parenting styles differed between parents and offspring gender. Offspring perceptions of a lack of maternal acceptance was associated with heightened risk of DE a year later in females, but not males. However, this was an interaction effect as maternal acceptance was a mediator for the relationship between gastric symptoms and DE. In contrast, there were no statistically significant effects for paternal acceptance.	Moderate
Korotana et al., 2018	Longitudinal T0 *N* = 446 T1 *N* = 383 T2 *N* = 352	Canada	T0 *N* = 446 (466 female) Range = 11–17 years	Parental environmental questionnaire	Mother and father	Minnesota eating behavior survey	Longitudinal associations between daughter DE and the parent-child relationship were limited. However, reciprocal associations were found between adverse parenting styles and daughter DE (body dissatisfaction, weight preoccupation, binge eating, compensatory behavior) as reporting of DE symptoms were associated with maladaptive changes to parenting styles.	Strong
Krug et al. (2014)	Longitudinal Attrition N/A	Australia	*N* = 1,391 (684 female) *M* = 11.59 years; S.D. = 0.52; range = 11–13 years)	N/A	N/A	Eating Disorder Inventory (unspecified)	For female participants, low parental monitoring was significantly associated with bulimia. Further, reports of low parental warmth and monitoring were associated with increased risk of reporting all DE symptoms (body dissatisfaction, drive for thinness, and bulimia) for adolescent females. However, for males, no significant associations were found between any parenting styles and reported DE symptoms.	Moderate
Krug et al., 2016	Longitudinal Attrition N/A	Australia	*N* = 1,300 (667 female) Range = 15–16 years	ATP parenting practices questionnaire	1 parent (95% mother)	Eating Disorder Inventory (EDI-1) body dissatisfaction scale	There was limited evidence for a direct parental effect on youths’ DE attitudes and behaviors. For males, there were no significant associations between reported parenting styles and any indicator of DEB. For females, low parental warmth alone was associated with bulimic behaviors. Further, exposure to both low parental warmth and monitoring increased females’ odds of reporting all DE outcomes (drive for thinness, bulimia, and body dissatisfaction).	Moderate
McEwen and Flouri, 2009	Cross-sectional	United Kingdom	*N* = 203 (125 female) *M* = 14.04 years; S.D. = 1.91; range = 11–18 years	Parental bonding instrument parental control scales	Father	Eating attitudes test (EAT-26)	Paternal overprotection, warmth, psychological control contributed independently to youth DE symptoms. Further, paternal psychological control and overprotection were directly associated with adolescent DE. However, paternal behavioral control (monitoring, knowledge, discipline) was not related to DE symptoms.	Strong
Meesters et al., 2007	Cross-sectional	Netherlands	*N* = 405 (224 female) *M* = 12.5; S.D. = 1.5; range = 10–16 years	Egna Minnen Beträffande Uppfrostran, (Swedish “My memories of upbringing”)	Mother and father	Children’s Eating Attitudes Test (ChEAT)	Reported maternal rejection was positively related to food preoccupation and dieting for both male and female participants. However, gender differences were observed as familial influences were more predictive of DE in boys than girls. High levels of both maternal and paternal control associated with dieting, food and muscle preoccupation in males. Further, maternal control significantly predicted muscle preoccupation in males.	Moderate
Pearson et al., 2009	Cross-sectional	United Kingdom	*N* = 328 (142 female) Range = 12–16 years Divided into younger participants *M* = 13.3 years, and older participants *M* = 15.6 years	Parenting style measure made by Kremers et al. (2003)	Converged	Youth/adolescent food frequency questionnaire	No significant interactions between parental involvement or strictness and any dietary behaviors were found after controlling for gender and age.	Strong
Rozenblat et al., 2017	Cross-sectional	Australia	Study 1. *N* = 650 (338 female) Range = 15–16 years Study 2. *N* = 304 (161 female)	Australian temperament project parenting practices scale Iowa family interaction rating scale	1 parent (mainly mother, no statistic available)	Eating Disorder Inventory (EDI-2)	Study 1. Bulimic symptoms were significantly associated with self-reported parental warmth and use of harsh punishment, though drive for thinness was not. Study 2. However, observations of parental hostility did not directly predict either bulimia or drive for thinness.	Weak
Zubatsky et al., 2015	Longitudinal attrition N/A	Switzerland	*N* = 2,516 (1,386 female) Divided into middle school, *M* = 12.8 years; S.D. = 0.8, at time 1, and *M* = 17.2 years; S.D = 0.6, at time 2 And high school, *M* = 15.8 years; S.D. = 0.8 at time 1; *M* = 20.4; S.D. = 0.8, at time 2	Created for study (assessing authoritative, authoritarian, permissive, and neglectful parenting)	Mother and father	Created for study (binge eating, WCBs, extreme WCBs)	The mother-youth relationship was significant, as maternal parenting style longitudinally predicted DEB (WCB) for both males and females. Specifically, youths with authoritarian mothers were more likely to have unhealthy WCBs compared to alternative parenting styles. For females, maternal authoritarian parenting style predicted increased risk for binge-eating behaviors; for males, less extreme WCB. In contrast, there were no significant associations between paternal parenting style and included youth DE symptoms.	Moderate

*DEB, disordered eating behaviors; DE, disordered eating; and WCB, weight control behaviors.*

### Quality and Risk of Bias Assessment

A modified version of the Effective Public Health Practice Project (EPHPP) Quality Assessment Tool for Quantitative Studies (Effective Public Health Practice and Project, 1998) was used to evaluate study quality and risk of bias. The following main domains were assessed: selection bias; study design; confounders; data collection methods; withdrawals and drop-outs; and appropriacy of analysis (“blinding” and “intervention integrity” were not relevant for the scope of this review). Included studies’ final score following quality appraisal were graded as “weak,” “moderate,” or “strong” (see Table 1). Studies with domains with ≥2, 1, and 0 weak ratings were considered to demonstrate “weak,” “moderate,” and “strong” risk of bias, respectively.

## Results

Eight studies were cross-sectional and eight were longitudinal (Table 1). All studies used community samples to assess DE, though one also included non-symptomatic controls (Hautala et al., 2011). Included studies were predominantly conducted in English-speaking countries, though studies also originated from Portugal (Fonseca et al., 2002), Netherlands (Meesters et al., 2007), Finland (Hautala et al., 2011) and Switzerland (Zubatsky et al., 2015). Overall, studies inconsistently reported the psychometric properties of tools used, with only 6 studies providing information on both parenting style and DE measures (Blodgett Salafia et al., 2009; Hautala et al., 2011; Berge et al., 2014; Hochgraf et al., 2017; Rozenblat et al., 2017; Korotana et al., 2018).

Parenting styles were assessed *via* offspring self-report and utilized a range of tools. Parental control scales were implemented in three studies, including the Psychological Control Scale Youth Report (Blodgett Salafia et al., 2007, 2009). The Parental Bonding Instrument (PBI) was implemented in a further three studies among samples including youths younger than the recommended 16 years of age (Furnham and Adam-Saib, 2000; McEwen and Flouri, 2009; Hautala et al., 2011).

DE outcomes were measured using self-report tools with the exception of one study, where parents reported on offspring DE using the Kiddie Schedule for Affective Disorders and Schizophrenia (Kerr et al., 2021). Of the rest, five studies used versions of the Eating Disorder Inventory (EDI-1 and -2) and two implemented the Eating Attitudes Test (EAT-26), with one study using a modified version for children (ChEAT, Meesters et al., 2007). The remaining six studies used various alternative tools.

Included studies used a range of statistical analysis techniques, though this information was missing in one study (Krug et al., 2014). Regression models were frequently used, with logistic, linear, and stepwise techniques used by four, two and one studies, respectively. Of the rest, four studies used structural equation modeling, of which one study utilized cross-lagged analyses (Korotana et al., 2018). Notably, structural equation modeling identified associations between parenting styles and youth DE were mediated by offspring emotional reactivity (Hochgraf et al., 2017), psychological distress (Blodgett Salafia et al., 2007) and lowered self-competence (Blodgett Salafia et al., 2009). The remaining four studies used various alternative analyses.

Overall, the divergence in the methodologies, measures and reporting in included studies limited the summarization and synthesis of findings within this scoping review.

### Cross-Sectional Studies

Both paternal (McEwen and Flouri, 2009) and maternal (Meesters et al., 2007) overprotection were correlated with DE food preoccupation. Further, youths who were exposed to controlling parenting styles presented with various DE symptoms, such as internalized muscle preoccupation (Meesters et al., 2007) and externalizing extreme WCBs (Berge et al., 2014). McEwen and Flouri (2009) assessed associations between paternal parenting styles and self-reported DE with additional predictor variables. Paternal psychological control and overprotection were directly associated with all selected indicators of DE behaviors for youths (McEwen and Flouri, 2009). However, conflicting evidence was found as no significant associations were found between any parenting styles and youth dietary behaviors after controlling for a number of potential confounders (Pearson et al., 2009).

### Longitudinal Studies

Similar to the cross-sectional results, adverse parenting presenting varying levels of responsiveness and demandingness was associated with a substantial range of operationalizations of DE. High levels of parental control predicted unhealthy WCBs (Zubatsky et al., 2015) and body dissatisfaction (Blodgett Salafia et al., 2007). Additionally, unresponsive parenting styles (exhibiting low behavioral monitoring) predicted various DE among daughters, such as body dissatisfaction (Blodgett Salafia et al., 2007) and bulimic behaviors (Krug et al., 2014). However, findings were inconsistent as many studies reported non-significant results for males which were not present for their female counterparts (Krug et al., 2014, 2016; Zubatsky et al., 2015; Kerr et al., 2021). Further, some studies compared longitudinal measurements of parental responsiveness and demandingness using logistic regression analyses (Hautala et al., 2011; Krug et al., 2016). Combined (Krug et al., 2016) and prolonged (Hautala et al., 2011) exposure to low parental warmth *and* high control predicted increased odds of, and more severe, DE. Two studies also aggregated findings at multiple time points throughout the study (Hautala et al., 2011; Korotana et al., 2018). Associations between parenting style and youths’ DE were dynamic: cross-lagged analyses suggested they were reciprocal (Korotana et al., 2018) and differed with age as associations were stronger in early teens relative to later teenage years (Hautala et al., 2011). Finally, the quality of included studies was restricted by a failure to report attrition rates, with only three studies including this information (Hautala et al., 2011; Korotana et al., 2018; Kerr et al., 2021).

## Discussion

The present review focused on associations between parenting styles and DE presentation in youth. Most studies provided preliminary support for an association between symptoms of DE and experience of various adverse parenting styles (e.g., McEwen and Flouri, 2009; Kerr et al., 2021), although some studies did not (e.g., Fonseca et al., 2002; Korotana et al., 2018). This resonates with previous work that has identified an association between DE beyond youth and other forms of non-abusive ALE present in family contexts, such as family dynamics (Mousoulidou et al., 2019). However, overall, review findings lacked consistency and stability, and associations were often indirect.

A number of characteristics of adverse parenting styles were associated with youth DE. Parenting styles that were perceived as controlling were associated with a range of youth DE outcomes (Meesters et al., 2007; McEwen and Flouri, 2009; Berge et al., 2014). Specifically, paternal overprotection correlated with (McEwen and Flouri, 2009) and increased risk of Hautala et al. (2011) DE symptoms. In addition, parental warmth was salient to youth DE, with exposure to low levels associated with a range of DE symptoms (Krug et al., 2014), including bulimia outcomes (Krug et al., 2016).

However, many studies found indirect effects of adverse parenting styles on DE; only one study established a direct association between high paternal demandingness, low responsiveness and DE (McEwen and Flouri, 2009). Associations between adverse parenting styles were mediated by offspring psychological distress (Blodgett Salafia et al., 2007), lowered self-competence (Blodgett Salafia et al., 2009) and emotional reactivity (Hochgraf et al., 2017), thus indicating the affective functioning of young people is an important facilitatory mechanism of DE onset. Attachment theory may thus be useful as a model to explain indirect effects identified in this scoping review, with parenting styles distally influencing youth eating pathology through correlates of the attachment system, such as offspring emotional competency (Laible, 2007) and self-competence (de Minzi, 2010).

Furthermore, longitudinal studies found interactions between parenting styles and DE varied throughout youth (Hautala et al., 2011; Korotana et al., 2018). The teenage years are a transitionary period for the parent-child relationship as parental influence decreases in salience relative to other social influences, including peers (Branje, 2018). It is likely the significance of mediating factors increases as offspring age due to changes in the parent-child relationship (Albarello et al., 2018), and contradictory findings within the review may be attributed to differences in sample age ranges. In sum, longitudinal studies implementing transactional models of parental styles better capture the development of DE across youth life-stages.

### Methodological Divergence

Although many studies assessed and aggregated maternal and paternal parenting styles, some studies converged parental contributions into a unitary construct of parenting in measurement (Pearson et al., 2009) and analysis (Rozenblat et al., 2017). However, the concept of parenting styles has been clarified to acknowledge the coexistence of discrete styles within joint parenting contexts, such as co-parent households (Kuppens and Ceulemans, 2019). Notably, some studies used the marital status of youths’ biological parents as inclusion criteria (e.g., Blodgett Salafia et al., 2007). Aggregated evidence from the present review indicate maternal and paternal parenting styles from the same household can differ (Zubatsky et al., 2015) and additively interact (Krug et al., 2014, 2016). Therefore, parents’ discrete styles are not consistently captured in convergent concepts of parenting, meaning the utility of current findings is restricted.

A significant differentiator of study quality was researchers’ consideration of the confounding effects of co-variates of parenting style. Higher quality studies situated parenting styles in the broader family context, for example collecting self-reported parental marital status (Krug et al., 2016) and number of siblings (Pearson et al., 2009), which has been implicated in modifying parenting style (Fan and Chen, 2020). However, research inconsistently assessed covariates and thus current data were insufficient to interpret meaningful patterns of findings within the review. Additionally, assessments of DE frequently measured drives for thinness and bulimia and body dissatisfaction, though researchers selected various tools to do so. Higher quality studies generally implemented well validated measures, such as the EDI-1 and EAT-26. However, some studies compiled (Berge et al., 2014) or created new measures by selecting operationalizations of DE from existing tools, without providing sufficient justification for selection (e.g., Fonseca et al., 2002). Collectively, the included studies introduced heterogeneity into the outcome of interest, thereby reducing the ability to synthesize findings and draw symptom-specific conclusions from youth experiences of parenting styles. Standardization of methods (specifically the measurement of covariates of parenting style, DE outcome and parenting style) is required to strengthen comparative conclusions drawn from the evidence base.

### Limitations

Only one study implemented a multi-modal procedure to assess parenting style (Rozenblat et al., 2017), thereby most studies in this review relied on an assumed convergence between knowledge and functioning (Baumrind, 2005; Herbers et al., 2017). Critically, observations of parenting style demonstrated low convergence with parental self-report (Rozenblat et al., 2017), suggesting conclusions relating to parenting style and youth DE outcomes may be dependent on the mode of assessment. Future research should implement standardized procedures incorporating multi-modal, aggregated assessments of maternal and paternal parenting styles.

Additionally, directional influences of parenting styles cannot be inferred from reported DE symptoms in cross-sectional studies. Though Baumrind (1991) conceptualizes parental influence on offspring outcomes as unidirectional, parenting style has been reconceptualized as bidirectional (Estlein, 2021) and reciprocal (Pinquart and Gerke, 2019), acknowledging the contribution of the offspring in parent-child interactions. Longitudinal evidence from the review indicates that DE acts a risk factor for adverse parenting, suggesting that youths’ DE symptoms subsequently modify parenting styles (Korotana et al., 2018). Establishing the directionality of associations is critical for the accurate interpretation of findings, and future research must continue to implement this design in order to capture the bidirectional and temporally dynamic nature of associations between parenting styles and youth DE throughout offspring development.

Finally, selection bias may be present as review results were limited to the English language. However, as the majority studies were conducted by English speaking countries, the risk of excluding additional relevant findings is anticipated to be low.

### Implications

Findings from the present review have implications for clinical practice. The prevalence of reported DE symptoms in youth across studies demonstrates the clinical necessity of intervention into maladaptive parenting styles. Clinicians must implement a patient-centered recovery model (e.g., Wetzler et al., 2020) and consider parental influences in the treatment of DE; family level interventions must be offered if appropriate to the young person’s experiences of DE (see Lock and Le Grange, 2019 for overview). From the findings of the present review, interventions into parenting styles characterized by controlling (e.g., Berge et al., 2014), overprotective (e.g., McEwen and Flouri, 2009) and unresponsive (e.g., Krug et al., 2016) parental behaviors are recommended. Such interventions may thus prevent the maintenance of youth DE symptoms. In addition, interventive promotion of “good enough” parenting styles exhibiting age appropriate control and responsiveness are recommended to remit youth from DE (Hautala et al., 2011, p.961). Subsequently, consistent with the conceptualization of maladaptive eating behaviors as a continuum from DE to an ED (Dinkler et al., 2021), targeted interventions into parental pathways of risk for the onset of DE can work to reduce family based risk of development and circumvent the development of subclinical symptoms into an ED (Stice and Van Ryzin, 2019).

## Conclusion

This rapid scoping review evaluated 16 studies to assess whether parenting style could be a risk factor for youth DE. Exposure to demanding, unresponsive, or a combination of these, parenting styles both predicted and correlated with reports of various symptoms of DE. However, included results were often conflicted, and comparative conclusions concerning the influence of adverse parenting styles on youth DE are limited due to the heterogenous operationalization and measurement of parenting styles, family context, and DE outcomes. Therefore, the present review does not establish a definitive account of the etiological influence of parental style to the onset of DE in youth. Further research implementing longitudinal and standardized procedures is essential for good quality research into parenting styles.

## Author Contributions

CH: conceptualization, methodology, investigation, and writing – original draft. BM: writing – review and editing. SD: conceptualization and writing – review and editing. All authors contributed to the article and approved the submitted version.

## Conflict of Interest

The authors declare that the research was conducted in the absence of any commercial or financial relationships that could be construed as a potential conflict of interest.

## Publisher’s Note

All claims expressed in this article are solely those of the authors and do not necessarily represent those of their affiliated organizations, or those of the publisher, the editors and the reviewers. Any product that may be evaluated in this article, or claim that may be made by its manufacturer, is not guaranteed or endorsed by the publisher.

## Appendix

Search strings:

***PsycArticles***
  Parenting styles or parenting or parental practices.
  Disordered eating or eating pathology or maladaptive eating.
  Adolescents or teenagers or children or kids or youth.
***PsycInfo***
  Parenting styles or parenting or parental practices.
  Disordered eating or maladaptive eating or eating pathology.
  Adolescents or teenagers or children or teen or youth.
***BASE***
  Parenting styles or parenting.
  Disordered eating or eating pathology or maladaptive eating.
  Teenagers or adolescents or children.
***CINAHL***
  Parenting styles or parenting.
  Disordered eating or maladaptive eating or eating pathology.
